# Capsaicin triggers autophagic cell survival which drives epithelial mesenchymal transition and chemoresistance in bladder cancer cells in an Hedgehog-dependent manner

**DOI:** 10.18632/oncotarget.10326

**Published:** 2016-06-29

**Authors:** Consuelo Amantini, Maria Beatrice Morelli, Massimo Nabissi, Claudio Cardinali, Matteo Santoni, Angela Gismondi, Giorgio Santoni

**Affiliations:** ^1^ School of Biosciences and Veterinary Medicine, University of Camerino, Camerino, Italy; ^2^ School of Pharmacy, Experimental Medicine Section, University of Camerino, Camerino, Italy; ^3^ Department of Molecular Medicine, Sapienza University, Rome, Italy; ^4^ Department of Medical Oncology, Polytechnic University of Marche, Ancona, Italy

**Keywords:** capsaicin, bladder cancer, autophagy, EMT, Hedgehog pathway

## Abstract

Bladder cancer (BC) is a common urologic tumor characterized by high risk of recurrence and mortality. Capsaicin (CPS), used as an intravesical drug for overactive bladder, was demonstrated to induce cell death in different cancer cells including BC cells.

Here we found that treatment of high-grade BC cells with high dose of CPS triggers autophagy. Infact, the CPS treatment alters the redox homeostasis by inducing production of radicals, mitochondrial depolarization, alterations of ADP/ATP ratio and activation of AMPK pathway stimulating the autophagic process in BC cells. The inhibition of autophagy, by using the specific inhibitor bafilomycin A or Beclin 1 knock-down, enhanced the CPS-induced cell death, demonstrating that CPS-induced autophagy acts as a pro-survival process in BC cells. By using PCR arrays and FACS analysis, we found that the CPS-treated BC cells displayed typical mesenchymal features of the epithelial mesenchymal transition (EMT) as elongated shape and over-expression of vimentin, α_5_ and β_1_ integrin subunits, integrin-like kinase and the anti-apoptotic Bcl-2 proteins. Moreover, we demonstrated that CPS treatment stimulates upregulation of Dhh/Ptch2/Zeb2 members of the Hedgehog signaling pathway, increases CD24, VEGFA and TIMP1 and decreases CD44 and ALCAM mRNA expression levels. By PTCH2 knock-down we found that the Hedgehog signaling pathway is involved in the CPS-induced autophagy and EMT phenotype.

Finally, we also showed that the CPS-resistant EMT-positive BC cells displayed an increased drug-resistance to the cytotoxic effects of mitomycin C, gemcitabine and doxorubicine drugs commonly used in BC therapy.

## INTRODUCTION

Bladder cancer (BC), characterized by high risk of recurrence and mortality, is among the fifth most common malignancies in the world. The majority of BC is represented by urothelial carcinoma (UC), also known as transitional cell carcinoma (TCC), while squamous and adenocarcinomas are diffused in a small percentage [1]. Although BC is considered to be responsive to the chemotherapy regiments, only few patients respond to single-agent therapy. For this reason, the finding of innovative anti-cancer combinations and new anti-neoplastic drugs is necessary.

Capsaicin (CPS), mainly known as food additive, is the active alkaloid found primarily in the chili peppers of the plant genus *Capsicum*, responsible for the hot pungent taste of these fruits. CPS, its derivatives and related compounds form a chemical group called capsaicinoids, known as exciting pharmacological agents for their ability to exert various effects on human body and in different diseases such as obesity, cardiovascular, gastrointestinal, dermatologic conditions and neurogenic overactive bladder [2]. In the last decades, several reports have demonstrated that CPS treatment is able to induce both pro- and anti-carcinogenic effects [3]. In fact, CPS inhibits cell proliferation and induces cell death in a TRPV1-dependent and independent manner in many different malignant human cell lines [4–6], including prostate and BC cells [7–9]; on the contrary, CPS promotes the carcinogenesis of colon, gastric and skin cancers stimulating cell proliferation and migration [2].

Autophagy is a self-degradative cellular mechanism used to degrade and recycle cytoplasmic components to provide energy during starvation, stress conditions or growth factor withdrawal promoting cell survival; however, excessive autophagy may also trigger autophagic-associated cell death [10]. It has been shown that CPS is able to stimulate autophagy in breast cancer and osteosarcoma cell lines. The CPS-induced autophagy is evoked at the same time with cell death and involves the DNA repair system functioning as a pathway that counteracts the CPS-induced apoptosis prolonging cancer cell survival [11, 12]. Moreover, several reports demonstrated that autophagy inhibition sensitizes BC cells to chemotherapy, indicating that in BC, targeting autophagy may be an effective therapeutic strategy to overcome drug resistance [13].

Novel evidence suggests that drug resistance is acquired by BC cells that undergo epithelial mesenchymal transition (EMT). EMT, characterized by morphological and molecular changes of cancer cells with increase of mesenchymal-related proteins, plays a pivotal role in the acquisition of malignant features such as invasion, metastasis and chemoresistance [14, 15]. In this regard, CPS induces EMT in colon cancer cells by modulating the reactive oxygen species (ROS) production and the AKT/mTOR pathways [16].

In BC, the tumorigenicity and EMT process are regulated through constitutive activation of different members of the Hedgehog (Hh) signaling pathway [17, 18]. Three Hh genes have been described in mammals: Sonic (SHH), Indian (IHH) and Desert (DHH). The Hh proteins are ligands for the patched receptor (Ptch), which negatively regulates smoothened protein (Smo). The Ptch binds to the Hh proteins, resulting in Ptch internalization in endosomes and lifting Ptch-mediated repression. Two homologous Ptch receptors, Ptch1 and Ptch2, have been described, both of which are able to interact with the Hh ligands [19].

Herein, the ability of CPS to induce autophagic cell survival, EMT and chemoresistance by involving the Hedgehog pathway was investigated in BC cell lines.

## RESULTS

### CPS induces autophagy in BC cells

**Figure 1:**
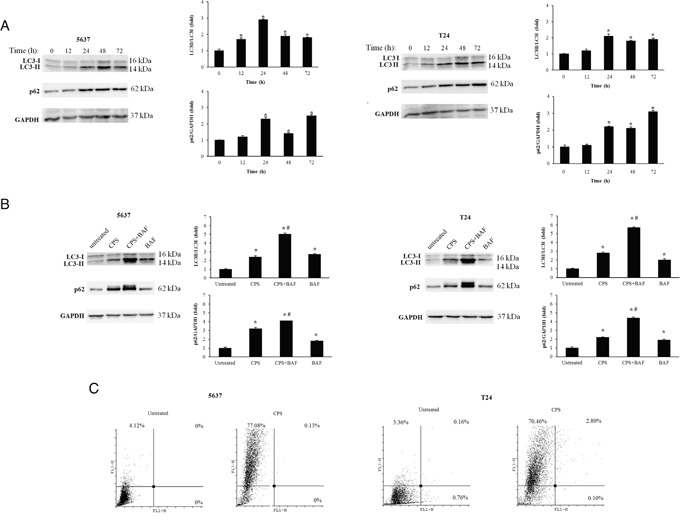
The CPS treatment triggers autophagy in BC cells **A.** Lysates from BC cells, untreated or treated for different times with CPS (300 μM) were separated on 14% or 8% SDS-PAGE and probed with anti-LC3 and anti-p62 Abs respectively. GAPDH protein levels were evaluated as loading control. Cropped blots are representative of one out of three separate experiments. Bars represent the densitometric analysis, shown as the mean ± SD of three different experiments, evaluated using untreated cells as calibrator. *p< 0.01 CPS-treated cells vs untreated. **B.** Lysates from BC cells, untreated or treated for 12 h with CPS (300 μM) alone or in combination with BAF (25 nM) were separated on 14% or 8% SDS-PAGE and probed with anti-LC3 and anti-p62 Abs respectively. GAPDH protein levels were used as loading control. Cropped blots are representative of one of three separate experiments. Bars represent the densitometric analysis, shown as the mean ± SD of three different experiments, evaluated using untreated cells as calibrator. *p< 0.01 vs untreated; #p<0.01 CPS+BAF- vs CPS or BAF-treated cells. **C.** AVOs were evaluated by AO staining and cytofluorimentric analysis in BC cells untreated or treated with CPS (300 μM) for 48 h. Dot plots are representative of one out of three separate experiments.

The activation of autophagy in CPS (300 μM)-treated BC cells was evaluated by assessing the processing of LC3 protein by western blot analysis. To this purpose, 5637 and T24 cancer cells, treated or not with CPS for different times, were analyzed for LC3 mobility. A significant accumulation of LC3-II proteins, starting at 12 h after treatment and sustained until 72 h was observed in both CPS-treated BC cell lines (Figure 1A). Similar results were obtained treating BC cells with rapamycin used as a positive control (data not shown). In addition, enhanced protein expression of sequestosome 1 (p62/SQSTM1), involved in the delivery of autophagic substrates and nucleation of autophagosomes was observed by western blot analysis in CPS-treated BC cells (Figure 1A). Since some autophagy inducers exert their effects both at p62 synthesis and degradation levels [20], p62 levels were also evaluated in BC cells treated with CPS in combination with the lysosomial inhibitor bafilomycin A (BAF, 25 nM). As shown in Figure 1B, BAF increased the p62 level in CPS-treated cells demonstrating the ability of CPS to induce an autophagic flux in BC cells. Similar results were obtained for LC3-II levels (Figure 1B). The CPS-induced autophagy was further confirmed by acridine orange (AO) staining and FACS analysis. A marked formation of acidic vesicular organelles (AVOs), a morphological characteristic of the autophagic process, was observed in CPS-treated BC cells respect to untreated cells (Figure 1C).

### CPS induces oxidative stress that triggers autophagy in BC cells

**Figure 2:**
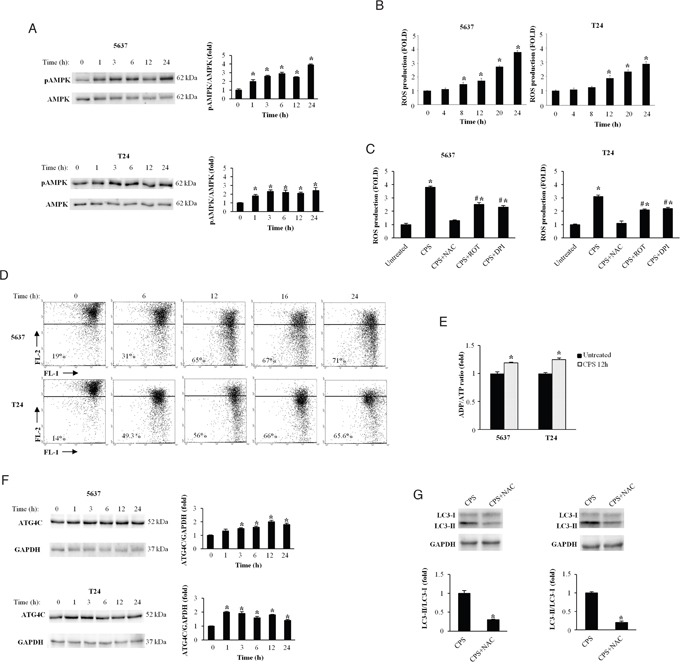
CPS triggers ROS production altering the mitochondrial redox homeostasis in BC cells **A.** Lysates from BC cells, untreated or treated for different times with CPS (300 μM) were separated on 10% SDS-PAGE and probed with anti-pAMPK mAb and anti-AMPK Ab. Cropped blots are representative of one of three separate experiments. Bars represent the densitometric analysis, shown as the mean ± SD of three different experiments, evaluated using untreated cells as calibrator. *p<0.01 CPS-treated vs untreated cells. **B.** ROS production was evaluated by DCFDA staining and cytofluorimetric analysis in 5637 and T24 cells treated for different times with CPS (300 μM). Data, shown as the mean ± SD of three independent experiments, are expressed as fold change with respect of ROS basal level. *p<0.01, CPS-treated vs untreated cells. **C.** ROS production was evaluated as above described in BC cells treated for 24h with CPS (300 μM) alone or in combination with NAC (10 mM), ROT (1 μM) or DPI (0.1 μM). Data, shown as the mean ± SD of three independent experiments, are expressed as fold change with respect of ROS basal level. *p<0.01 CPS-, CPS+ROT or CPS+DPI-treated vs untreated cells; #p<0.01 CPS+ROT or CPS+DPI-treated vs CPS-treated cells. **D.** Time course analysis of ΔΨm changes in 5637 and T24 cells untreated or treated for different times with CPS (300 μM), was evaluated by JC-1 staining and biparametric FL1 (green)/FL2(red) flow cytometric analysis. Numbers indicate the percentage of cells showing a drop in ΔΨm-related red fluorescence intensity. Data are representative of one out of three separate experiments. **E.** ADP/ATP ratio in T24 and 5637 cells, untreated or treated with CPS (300 μM) for 12 h, was evaluated by bioluminescent assay. Data are the mean ± SD of three independent experiments. *p<0.01 CPS-treated vs untreated cells. **F.** Lysates from BC cells, treated as described in panel A, were separated on 10% SDS-PAGE and probed with Atg4C or anti-GAPDH Abs. Cropped blots are representative of one of three separate experiments. Bars represent the densitometric analysis, shown as the mean ± SD of three different experiments, evaluated using untreated cells as calibrator *p<0.01 CPS-treated vs untreated cells. **G.** Lysates from BC cells, treated for 72 h with CPS (300 μM) alone or in combination with NAC (10 mM) were separated on 14% SDS-PAGE and probed with anti-LC3 Ab. GAPDH protein levels were used as loading control. Bars represent the densitometric analysis, shown as the mean ± SD of three different experiments, evaluated using CPS-treated cells as calibrator. *p<0.01 CPS+NAC- vs CPS-treated cells.

The energy sensor AMPK acts as a major regulator of cellular ATP levels and protects cells against stresses. Recent evidence demonstrated that activation of AMPK by calcium and ROS signalling inhibits the mTOR complex and induces autophagy [21]. Thus, we evaluated the AMPK activation by measuring its phosphorylation status (pAMPK), reactive oxygen species (ROS) generation, the mitochondrial depolarization and ATP levels in untreated and CPS (300 μM)-treated BC cells. Time-course immunoblot analysis showed that enhancement of pAMPK was evident at 1 h, peaked at 3 and 6 h after CPS exposure, remaining sustained at later time-points (Figure 2A).

ROS signals and mitochondrial alterations have been associated to vanilloid-mediated effects [22]. Thus, the involvement of ROS production during stress-induced autophagy was evaluated in CPS-treated BC cells by cytofluorimetric analysis. We found that CPS treatment induces ROS generation in BC cells, with a sustained accumulation which begins at 8 h and increases at later time-points (Figure 2B). The ROS scavenger N acetylcysteine (NAC, 10 mM) completely inhibited ROS production, indicating that CPS stimulates peroxide accumulation in BC cells (Figure 2C). Moreover, to better assess the source of CPS-induced ROS generation, BC cells were treated with CPS alone or in combination with the mitochondrial respiratory chain inhibitor rotenone (ROT, 1 μM) or the NAD(P)H oxidase inhibitor diphenyleneiodonium (DPI, 0.1 μM). As shown in Figure 2C, ROT and DPI markedly reduced ROS production indicating that CPS treatment affects both the mitochondrial respiration and the membrane-bound enzyme complex (Figure 2C). Since mitochondrial dysfunction is often associated with ROS generation, we used JC-1 labeling and cytofluorimeter to analyze ΔΨm in 5637 and T24 BC cells. We found that treatment with CPS induces a time-dependent decrease of red fluorescence (depolarization). The CPS-induced ΔΨm dissipation was evident at 6 h, increased at 12 h and remained sustained at 24 h (Figure 2D). Changes in ADP/ATP ratio are indicative of alterations in energy metabolism. By using bioluminescent assay, we also demonstrated that the ADP/ATP ratio is increased in CPS-treated as compared with untreated cells (Figure 2E).

The cysteine protease Atg4C, regulated by ROS generation, is involved in the formation and maturation of autophagosomes and represents the main cellular target of oxidative signals in CPS-induced autophagy [23]. Herein we found that CPS increases at 1-6 and 12-24 h the expression of Atg4C protein in T24 and 5637 BC cells, respectively (Figure 2F). Finally, by western blot analysis, we demonstrated that the ROS inhibitor, NAC (10 mM) reverted the CPS-induced autophagy (Figure 2G). NAC alone did not induce changes in the LC3 levels respect to untreated cells (Supplementary Figure 1A). Overall, our data demonstrated in BC cells that CPS treatment alters the mitochondrial redox homeostasis and induces autophagy in a ROS dependent manner.

Finally to evaluate the effects of CPS on autophagic gene pathway, we performed a RT Profiler array comparing untreated with CPS-treated BC cells at 12 h after treatment. We found that GABARAPL1, MAP1LC3B, SQSTM1, IRGM, ULK1, TNF and PTEN genes mediating different steps of the autophagic process were strongly upregulated and FADD and TP73, involved in the apoptotic pathway and IGF1, implicated in cell proliferation, were downregulated (Table 1).

**Table 1:** CPS stimulates the autophagic signaling pathway in BC cells

Gene Bank ID	Symbol	Description	Fold change	
			5637 cell line	T24 cell line
NM_003824	FADD	Fas (TNFRSF6)-associated via death domain	−2.01	−1.78
**NM_031412**	**GABARAPL1**	**GABA(A) receptor-associated protein like 1**	**3.59**	**1.74**
NM_001017963	HSP90AA1	Heat shock protein 90kDa alpha (cytosolic), class A member 1	−1.13	**2.31**
NM_000618	IGF1	Insulin-like growth factor 1 (somatomedin C)	−12.77	−3.07
**NM_001145805**	**IRGM**	**Immunity-related GTPase family, M**	**3.28**	**1.13**
**NM_022818**	**MAP1LC3B**	**Microtubule-associated protein 1 light chain 3 beta**	**2.20**	**2.69**
**NM_000314**	**PTEN**	**Phosphatase and tensin homolog**	**4.10**	**1.13**
**NM_003900**	**SQSTM1**	**Sequestosome 1**	**2.69**	**1.79**
**NM_000594**	**TNF**	**Tumor necrosis factor**	**7.54**	**8.34**
**NM_003565**	**ULK1**	**Unc-51-like kinase 1 (C. elegans)**	**2.52**	**1.41**
NM_005427	TP73	Tumor protein p73	−1.74	−2.17

Autophagy RT profiler PCR array in mRNA samples extracted from 5637 and T24 BC cells treated for 12 h with vehicle or CPS (300 μM). Values represent fold differences of individual gene expression in CPS-compared to vehicle-treated BC cells. The expression levels were normalized to the average Ct value of two housekeeping genes (GAPDH and RPLP0) and calculated by the ΔΔCt method. In bold up-regulated genes. Data shown are representative of one out of three separate experiments.

### CPS-induced autophagy acts as pro-survival mechanism in BC cells

**Figure 3:**
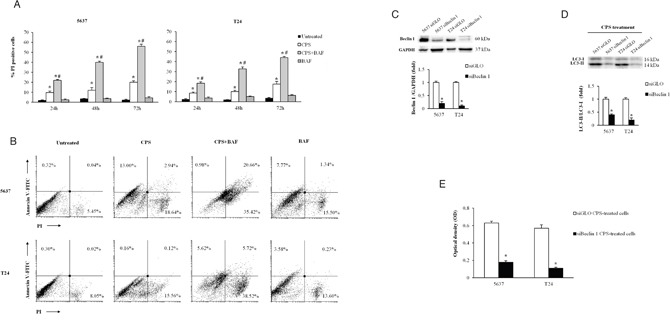
The CPS-induced autophagy acts as a pro-survival process in BC cells **A.** Cell death was evaluated in BC cells, untreated or treated for different times with CPS (300 μM) alone or in combination with BAF (25 nM) by FACS analysis. Data shown are the mean ± SD of three independent experiments. *p<0.01 vs untreated; #p<0.01 CPS plus BAF vs CPS- or BAF-treated cells. **B.** Cell death type was determined by Annexin V/PI staining and cytofluorimetric analysis in BC cells after 72 h of treatment with CPS (300 μM) alone or in combination with BAF (25 nM). Data are representative of one out of three separate experiments. Numbers represent the percentage of positive cells. **C.** Lysates from siGLO and siBeclin 1 BC cells were separated on 8% SDS-PAGE and probed with anti-Beclin 1 Ab. Cropped blots are representative of one of three separate experiments. Bars represent the densitometric analysis, shown as the mean ± SD of three different experiments, evaluated using siGLO cells as calibrator. *p<0.01 siBeclin 1 vs siGLO cells. **D.** Lysates from siGLO and siBeclin 1 BC cells treated for 72 h with CPS (300 μM) were separated on 14% SDS-PAGE and probed with anti-LC3 Ab. Cropped blots are representative of one of three separate experiments. Bars represent the densitometric analysis, shown as the mean ± SD of three different experiments, evaluated using siGLO cells as calibrator. *p<0.01 CPS-treated siBeclin 1 vs CPS-treated siGLO cells. **E.** Cell viability was assessed by MTT assay in siGLO and siBeclin 1 BC cells treated for 72 h with CPS (300 μM). Data shown are the mean ± SD of three independent experiments. *p<0.01 siBeclin 1 vs siGLO CPS-treated BC cells.

In cancer, autophagy is generally thought to play a pro-survival role allowing cancer cells to survive during metabolic stress; however excessive autophagy can also induce cell death [10]. To better understand the role of CPS-induced autophagy, we evaluated, by cytofuorimetric analysis, cell death/survival in BC cells treated for different times with CPS (300 μM) in the presence or not of the autophagic inhibitor BAF (25 nM). By PI and annexin V staining, we found that CPS induces necrotic cell death in a time-dependent manner in 5637 and T24 BC cells, and that BAF enhances the CPS-induced cytotoxic effects (Figure 3A and 3B).

Moreover, since a pivotal role of Atg6/Beclin-1 in autophagy, we knocked-down Beclin 1 gene (siBeclin 1) in BC cells. As shown in Figure 3C, Beclin 1 protein expression was strongly down regulated in siBeclin 1, compared with control-BC cells (siGLO). The siBeclin 1 and siGLO BC cells were treated with CPS for 72 h and cell growth was evaluated by MTT assay. Interestingly, knock-down of Beclin 1, that abrogates CPS-induced autophagy (Figure 3D), strongly reduced BC cell growth (Figure 3E). Overall, these results demonstrated a survival role for CPS-induced autophagy in BC cells.

### CPS-induced autophagy triggers epithelial mesenchymal transition (EMT) in BC cells

**Figure 4:**
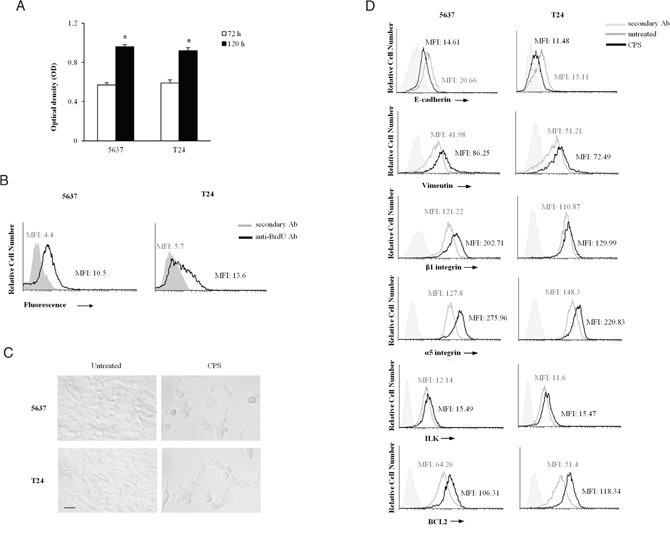
The CPS-resistant BC cells display the EMT phenotype **A.** Cell growth was assessed by MTT assay in 5637 and T24 BC cells treated for 72 h or 120 h with CPS (300 μM). Data shown are the mean ± SD of three independent experiments. *p<0.01 120 h CPS-treated vs 72 h CPS-treated BC cells. **B.** Cell replication was assessed by BrdU incorporation and FACS analysis in BC cells treated for 120 h with CPS (300 μM) and cultured in medium for additional 24 h. One representative out of three independent experiments is shown. Numbers indicate the Mean Fluorescence Intensity (MFI). **C.** Cell morphology was evaluated by light microscopy in BC cells, untreated or treated with CPS (300 μM) for 120 h. One representative out of three independent experiments is shown. Bar = 25 μM. **D.** FACS analysis was performed in BC cells, untreated or treated with CPS (300 μM) for 120 h, stained with anti-E cadherin, anti-vimentin, anti-β1 and anti-α5 integrin subunits, anti-ILK and anti-Bcl2 Abs followed by respective FITC-conjugated secondary Abs. One representative out of three independent experiments is shown. Numbers indicate the Mean Fluorescence Intensity (MFI).

Since the CPS-induced autophagy acts as a pro-survival process, BC cells were treated with CPS (300 μM) for 72 and 120 h to assess the proliferative capability by MTT and BrdU incorporation. Our results evidenced that CPS-resistant BC cells are actively replicating (Figure 4A and 4B), suggesting that they could be responsible for bladder cancer relapse.

Then, we better characterized the phenotype of CPS-resistant BC cells at 120 h after CPS treatment by light microscopy and cytofluorimetric analysis. As shown in Figure 4C and Supplementary Figure 1B, CPS-treated BC cells, displayed an increased cell size and morphological changes with a conversion from a “cuboidal” epithelial structure into an elongated mesenchymal shape, compared to untreated cells, suggesting that prolonged exposure to CPS stimulates the EMT process in BC cells. To further address the presence of the EMT phenomenon in CPS-resistant BC cells, we investigated the expression of markers typical of the epithelial mesenchymal transition. Our results showed that the expression of E-cadherin is significantly reduced in CPS-treated cells, respect to untreated BC cells, whereas vimentin, α_5_ and β_1_ integrin subunits, ILK and Bcl-2 are upregulated (Figure 4D), indicating the occurrence of EMT and the acquisition of a more aggressive phenotype in CPS-resistant BC cells.

### CPS induces autophagy and EMT in an Hedgehog signaling pathway dependent manner

The Hedgehog pathway has been found to regulate autophagy and EMT [24–26]. Thus, we first evaluated by quantitative real time PCR (qRT-PCR) the expression of different members of Hedgehog signaling pathway, Shh, Ihh and Dhh ligands, their specific receptors, Patched 1 and 2 (Ptch1 and Ptch2) and some related genes such as Zeb1, Zeb2, ALCAM and VEGFA in BC cells treated with CPS for 120 h. Upregulation of the Dhh/Ptch2/Zeb2 pathway as well as downregulation of Shh or Ihh/Ptch1/Zeb1 genes were observed in CPS-treated BC cells as respect to untreated cells. In addition, increased CD24, VEGFA and TIMP1 and decreased CD44 and ALCAM mRNA levels were found in both T24 and 5637 BC cell lines (Table 2).

**Table 2:** CPS treatment influences the Hedgehog and EMT pathways

Assay ID	Gene Bank ID	Symbol	Description	Fold change	
				5637 cell line	T24 cell line
**Hs03044178_g1**	**NM_013230**	**CD24**	**CD24**	**2.12**	**1.95**
Hs01075861_m1	NM_000610	CD44	CD44	−1.49	−1.58
Hs00179843_m1	NM_000193	SHH	Sonic Hedgehog	−2.97	−2.24
Hs00745531_s1	NM_002181	IHH	Indian Hedgehog	−7.89	−2.17
**Hs00368306_m1**	**NM_021044**	**DHH**	**Desert Hedgehog**	**25.02**	**3.93**
Hs00181117_m1	NM_000264	PTCH1	Patched 1	−1.72	−19.97
**Hs01085642_m1**	**NM_001166292**	**PTCH2**	**Patched 2**	**2.03**	**7.49**
Hs00977641_m1	NM_001243280	ALCAM	Activated leukocyte cell adhesion molecule	−2.00	−1.27
**Hs00900055_m1**	**NM_001025366**	**VEGFA**	**Vascular endothelial growth factor A**	**1.96**	**2.39**
**Hs00171558_m1**	**NM_003254**	**TIMP-1**	**TIMP metallopeptidase inhibitor 1**	**5.80**	**5.00**
Hs00232783_m1	NM_001128128	ZEB1	Zinc finger E-box binding homeobox 1	−1.33	−9.85
**Hs00207691_m1**	**NM_001171653**	**ZEB2**	**Zinc finger E-box binding homeobox 2**	**2.06**	**1.82**

Custom Taqman Assay in mRNA samples extracted from untreated and CPS-treated cells for 120 h. Values represent fold differences of individual gene expression in CPS treated compared to untreated BC cells. The expression levels were normalized to the average Ct value of two housekeeping genes (GAPDH and β actin) and calculated by the ΔΔCt method. In bold up-regulated genes. Data shown are representative of one out of three separate experiments.

**Figure 5:**
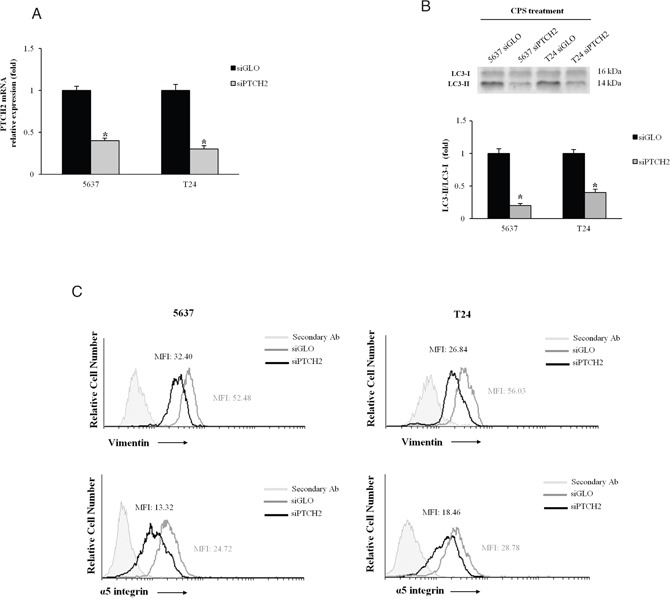
The Hedgehog signaling pathway is involved in the CPS-induced autophagy and EMT phenotype **A.** PTCH2 mRNA expression, normalized to GAPDH levels, was evaluated in siGLO and siPTCH2 BC cells by qRT-PCR.*p<0.01 siPTCH2 vs siGLO BC cells. **B.** Lysates from siGLO and siPTCH2 BC cells treated for 72 h with CPS (300 μM) were separated on 14% SDS-PAGE and probed with anti-LC3 Ab. Cropped blots are representative of one of three separate experiments. Bars represent the densitometric analysis, shown as the mean ± SD of three different experiments, evaluated using siGLO cells as calibrator. *p<0.01 CPS-treated siPTCH2 vs CPS-treated siGLO cells. **C.** FACS analysis was performed in siGLO and siPTCH2 BC cells, treated with CPS (300 μM) for 120 h, stained with anti-vimentin and anti-α5 integrin subunit Abs followed by respective FITC-conjugated secondary Abs. One representative out of three independent experiments is shown. Numbers indicate the Mean Fluorescence Intensity (MFI).

To better investigate the role of the Hedgehog pathway, BC cells were silenced for PTCH2 (siPTCH2). A reduction of PTCH2 mRNA was observed in siPTCH2 as respect to siGLO (control) BC cells (Figure 5A). Therefore, we treated silenced BC cells with CPS for 72 and 120 h to assess autophagy and EMT transition, respectively. Our results showed that the LC3II/LC3I ratio is reduced in siPTCH2 compared with siGLO BC cells (Figure 5B) and more interestingly, FACS analysis displayed that PTCH2 knockdown decreased the expression of vimentin and α_5_ integrin subunit in CPS-treated cells. Overall our findings demonstrated that the Hedgehog signaling pathway is involved in CPS-induced autophagy and EMT phenotype in BC cells.

### The EMT, induced by CPS, promotes chemotherapeutic drug-resistance of BC cells

**Figure 6:**
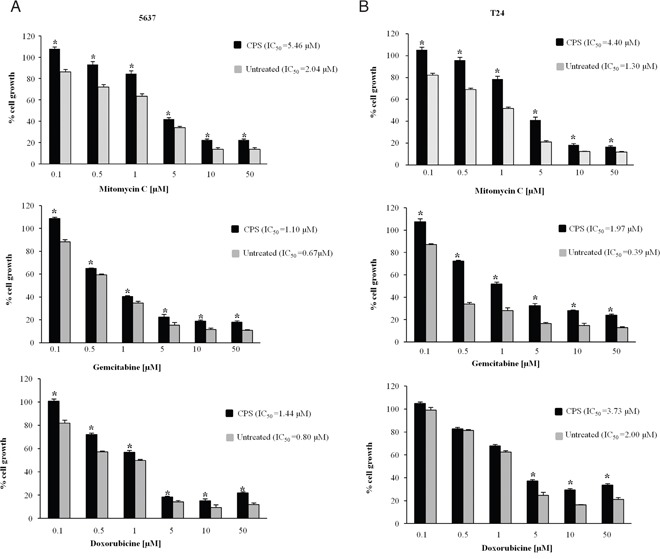
CPS-resistant BC cells characterized by EMT phenotype, show resistance to chemotherapeutic agents **A-B.** BC cells treated with CPS for 120 h were detached, counted and plated for additional 48 h in medium supplemented with different doses of mitomycin C, gemcitabine or doxorubicine. At the end of treatment MTT assays were performed. Data shown are the mean ± SE of three independent experiments. *p<0.01 CPS-treated vs untreated BC cells. IC_50_ was calculated using GraphPad.

Hedgehog signaling pathway activation is positively correlated with chemoresitance in cancer cells [24, 27]. Thus, we finally investigated the drug-sensitivity of CPS-induced EMT cells to chemotherapeutic agents commonly used in BC therapy. To this purpose, CPS-exposed (120 h) BC cells, were further treated for additional 48 h with different doses of mitomycin C, gemcitabine and doxorubicine. As shown in Figure 6A and 6B, the CPS-resistant BC cells were significantly less sensitive to chemotherapeutic agents compared with untreated cells. In fact the IC_50_ resulted about two-three fold higher for all tested drugs in CPS-treated BC cells respect to untreated cells.

Taken together, our results demonstrated that CPS-resistant 5637 and T24 BC cells showing the EMT phenotype display chemotherapeutic drug-resistance.

## DISCUSSION

Herein, we demonstrated that CPS, by triggering autophagic cell survival, stimulates BC cells to undergo epithelial mesenchymal transition and to develop chemotherapeutic drug-resistance through the Hedgehog signalling pathway.

In CPS-treated BC cells, cellular energy imbalance resulting from ROS generation, alterations of ATP levels, mitochondrial depolarization and AMPK signalling pathway activation, promotes autophagy as demonstrated by the enhancement of LC3-II/LC3-I ratio and accumulation of p62 protein evaluated in CPS plus BAF-treated BC cells. We also demonstrated by using the ROS inhibitor NAC that the intracellular ROS production, triggered by CPS in BC cells, is responsible for the oxidative stress-induced autophagy, that started at 12 h and remained sustained until 72 h. The time-dependent increase of p62 SQSTM1 protein expression was a consequence of enhanced transcription level as evidenced by qRT-PCR. Moreover, CPS treatment increased the expression of autophagic genes such as GABARAPL1 and MAP1LC3B associated with autophagic vesicles formation [28], IRGM and ULK1 engaged in the autophagy complex initiation [29], TNFα and PTEN, involved in autophagy induction [30, 31].

Autophagy promotes stress-induced cell survival or autophagic cell death [10]. In this regard, we showed that CPS-induced autophagy mediates a pro-survival effect in 5637 and T24 BC cells. In fact a marked increase of necrotic cells was evident in BC cells treated with CPS in combination with the autophagic inhibitor BAF. In addition, knockdown of Beclin 1, that inhibits the CPS-induced autophagy, reduces the growth of BC cells. Similarly, it has been recently demonstrated that CPS-induced autophagy rescues breast carcinoma and osteosarcoma cells from death [11, 12]. Taken together, our findings indicated that CPS-stimulated autophagy acts as a mechanism counteracting cell death and contributing to the survival of BC cells.

The EMT is characterized by morphological changes from epithelial to spindle-shaped mesenchymal like cells, increased cell size, loss of cell adhesion as consequence of changes in integrin expression, in particular α_5_ β_1_ overexpression [15], increased invasiveness and expression of mesenchymal markers especially vimentin [32, 33]. CPS has been found to induce EMT in colon cancer cells [16], but no data on BC has been provided so far. To better clarify the effect of CPS in 5637 and T24 BC cells, we characterized the phenotype of CPS-resistant BC cells rescued by autophagy. CPS-treated BC cells show increased cell size and mesenchymal-like morphology, enhanced expression of vimentin, α_5_ and β_1_ integrin subunits and integrin like kinase (ILK) and anti-apoptotic Bcl-2 proteins and downregulation of E-cadherin. The CPS-induced EMT-positive BC cells are actively replicating suggesting a possible involvement in bladder cancer relapse. In agreement with our results, it is well known that ILK activation is involved in survival, proliferation, motility, invasion, angiogenesis [34] and that ILK silencing inhibits EMT, cell growth and metastasis in BC cells [35]. In addition in BC, increased Bcl-2 proteins, that correlates with high pathologic stage, disease recurrence and mortality rate [36], may enhance the resistance to necrotic death and support the BC autophagic survival.

Activation of the Hedgehog pathway in human BC cells and its association with BC progression and clinical outcome have been recently reported [17, 37–39]. Herein, by qRT-PCR, we observed that exposure of BC cells to high dose of CPS for 120 h, modulates the expression of different members of the Hedgehog signaling pathway. Thus, upregulation of Dhh/Ptch2/Zeb2, as well as downregulation of Shh and/or Ihh/Ptch1/Zeb1 transcripts were found in CPS-treated BC cells, as respect to control cells. In addition, the silencing of PTCH2 gene in BC cells inhibited the CPS-induced autophagy and reverted the EMT phenotype, as evaluated by the reduction of LC3-II, vimentin and α_5_ integrin subunit. Hedgehog (Hh) signals acts as survival factor by maintaining the viability of UC cells and increased Hh activity is required for BC cell growth. Constitutive activation of different members (e.g. ligands and receptors) of the Hedgehog pathway highly correlates with BC progression [17]. Recent evidence in HeLa cells showed that Ptch2 overexpression, increased LC3-II levels and autophagy, in the absence and presence of BAF, and knockdown of PTCH2 reduced the LC3-II levels in basal and induced autophagy [40]. Increased PTCH2 mRNA expression correlates with poorer overall survival in muscle-invasive BC [37] and a functional link of PTCH2 in regulating the activation of Dhh pathway has been suggested [41]; moreover enhanced Dhh expression was associated with invasive prostate and bladder cancers and hormone-refractory behaviour [42]. In addition, overexpression of Zeb2, a transcription factor detected in infiltrating UC [43], that promotes the EMT [44] and regulates the expression of integrins [45], may be responsible for the increase of α_5_ integrin expression level in EMT-positive CPS-treated T24 and 5637 BC cells.

We also found increased expression of EMT-related genes such as CD24, VEGFA, TIMP1 and decreased levels of activated cell adhesion (ALCAM/CD166) and CD44 molecules in BC cell lines treated with CPS. The CD24 and CD44 antigens mark distinct cell population in BC [46], and CD24 expression has been associated with a more aggressive phenotype, reduced survival and poor prognosis [47]. Metalloproteases and their inhibitors, TIMPs, play a pivotal role in BC progression. TIMP1 overexpression correlates with pathological stage and poor prognosis in BC and induces the phenotypic changes linked with increased vimentin levels [48]. Furthermore, ALCAM loss/downregulation is associated with a more invasive phenotype and poorer outcome in BC [49]. Since the key-role of VEGFA in vimentin expression promoting EMT changes [50] and chemoresistance [51] in BC cells, VEGFA overexpression may be partially responsible for the acquisition of the EMT phenotype and drug resistance found in CPS-treated BC cells.

The EMT regulates drug-resistance and invasion in BC cells [36], and EMT prevention increases the BC sensitivity to anti-neoplastic agents [14]. The development of chemoresistance during BC treatment prejudices the effects of the chemotherapy agents and is responsible for the high morbidity and mortality of BC in the world [52]. Here, we found that CPS-induced EMT in BC cells results in an increased drug-resistance of BC cells to the cytotoxic effects of mitomycin C, gemcitabine and doxorubicine.

From a clinical point of view, it is now well established that the induction of autophagy interferes with the efficacy of cancer therapeutics [53]. In fact, recent studies reported increased chemoresistance in BC cells with concurrent up-regulation of autophagy related genes [54]. At this regard, at present, there is substantial evidence demonstrating the improved therapeutic effects by using the established cancer therapeutics agents in combination with autophagy inhibitors [53].

Overall, our results, showing that the escape of BC cells from CPS-induced death, by triggering the autophagic survival, Hedgehog pathway activation, EMT and chemotherapy drug resistance, are in line with these recent findings. Therefore the treatment of BC cells with CPS in combination with specific autophagic or EMT/Hedgehog pathway inhibitors, could represent an interesting new approach in BC therapy.

## MATERIALS AND METHODS

### Cell lines

5637 and T24 BC cell lines, purchased from Leibniz-Institute DSMZ (Braunschweig, Germany) on May 5, 2015 with authentication by DNA typing, were maintained in RPMI-1640 medium (Lonza Bioresearch, Basel, Switzerland) supplemented with 10% heat-inactivated fetal bovine serum, 2.5 mM N-2-hydroxyethylpiperazine-N'-2-ethanesulfonicacid (HEPES), 2 mM L-glutamine, 100 IU/ml of penicillin and 100 μg/ml of streptomycin (Lonza) at 37°C, 5% CO_2_ and 95% humidity.

### Reagents

Capsaicin (CPS, trans-8-methyl-N-vanillyl-6-nonenamide) was purchased from Sigma Aldrich (MO, USA). The following antibodies (Abs) were used: anti-p62, anti-AMPK, anti-pAMPK, anti-ATG4C and anti-Beclin 1 (1:1000, Cell Signaling Technology, CO, USA), anti-microtubule-associated protein-1ight chain 3 (LC3, 2 μg/ml, Novus Biologicals, CO, USA), horseradish peroxidase (HRP)-conjugated anti-glyceraldehyde-3-phosphate dehydrogenase (anti-GAPDH, 1:5000, Sigma Aldrich), anti-α_5_ integrin (1:25, Beckman Couture, FL, USA), anti-β_1_ integrin (1:50, Santa Cruz Biotechnology, CA, USA), anti-Bcl2 (1:50, Bethyl Laboratories Inc, TX, USA), anti-Integrin linked kinase (ILK, 1:50, Bethyl Laboratories Inc), anti-vimentin (1:50, Merk Millipore, MA, USA), anti-E cadherin (1:50, Santa Cruz Biotechnology), anti-BrdU (1:20, Becton Dickinson Biosciences, CA, USA). The following secondary Abs were used: HRP-conjugated donkey anti-rabbit (1:2000) and HRP-conjugated sheep anti-mouse (1:2000) from GE Healthcare Bio-Sciences (Uppsala, Sweden), FITC-conjugated anti-mouse Ab (1:40), FITC-conjugated anti-rabbit (1:40) and FITC-conjugated donkey anti-goat (1:40) Abs were from Santa Cruz Biotechnology. 5,5,6,6-tetrachloro-1,1,3,3-tetraethylbenzimidazolylcarbocyanine iodide (JC-1, 10 μg/ml) and Acridine Orange (AO 1 μg/ml) were from Invitrogen (CA, USA). 20,70-Dichlorofluorescein diacetate (DCFDA, 10 μg/ml), dimethyl sulfoxide (DMSO, used as vehicle), N acetylcysteine (NAC, 10 mM), Diphenyleneiodonium chloride (DPI, 0.1 μM), Rotenone (ROT, 1 μM), 5-bromo-2-deoxyuridine (BrdU), propidium iodide (PI, 2 μg/ml) and 3-(4,5-dimethylthiazol-2-yl)-2,5-diphenyltetrazolium bromide (MTT) were from Sigma Aldrich. Bafilomycin A1 (BAF, 25 nM) was from Labogen (Catania, Italy). Annexin V-FITC (5 μl/ml) was purchased from Enzo Life Sciences (NY, USA).

### Beclin 1 and PTCH2 gene silencing

siGENOME SMARTpools for Beclin 1 (siBeclin 1), siCONTROL nontargeting siRNA (siGLO) used as negative control were purchased from Thermo Scientific-Dharmacon (CO, USA). For gene silencing experiments, 5637 and T24 cells were plated in a six well plate at the density of 3 × 10^4^/ml and 160 pmol of siBeclin 1 or siGLO was added to the wells for 48 h, following the METAFECTENE SI PRO transfection protocol (Biontex Laboratories, CA, USA).

To knockdown PTCH2, the GeneSolution siRNA for PTCH2 (siPTCH2), negative control siRNA duplex (siGLO) and the HiPerfect Transfection reagent (Qiagen, CA, USA) were used following the Fast-forward Transfection protocol. Briefly 5637 and T24 cells were plated in a twenty-four well plate at the density of 5 × 10^4^/ml and siPTCH2 or siGLO at the concentration of 5nM were added to the wells for 48 h. Then silenced cells were detached, counted and plated for MTT, western blot and FACS analyses.

### MTT assay

The colorimetric MTT assay was used to evaluate cell viability. Briefly, 5637 and T24 BC cells (3×10^4^/ml) were seeded into 96-well plates and cultured with CPS (300 μM) for 72 and 120 h at 37°C, 5% CO_2_. At the end of treatment, 0.8 mg/ml of MTT was added to the samples and incubated for 3 h. Then the supernatants were discarded and coloured formazan crystals, dissolved with 100 μl/well of DMSO, were read by an enzyme-linked immunosorbent assay reader (BioTek Instruments, VT, USA). Four replicates were used for each treatment and data were represented as the average of at least three separate experiments. In some experiments, BC cells silenced or not for Beclin 1, were treated with CPS (300 μM) for 72 h before to perform the MTT assay. Finally to assess drug resistance, the assay was performed in BC cells, that after treatment with CPS for 120 h, were detached, counted, plated at 3×10^4^/ml in a 96 well plate and treated for additional 48 h with different doses (from 0.01 to 50 μM) of mitomycin C, gemcitabine and doxorubicine. The half maximal inhibitory concentration (IC_50_) was determined using GraphPad (GraphPad Software, CA, USA).

### Cell death analysis

To evaluate cell death, 5637 and T24 cells (3×10^4^/ml), untreated or treated for different times (24, 48 and 72 h) with CPS (300 μM) alone or in combination with BAF (25 nM), were stained with PI. To assess cell death type, BC cells treated for 72 h with CPS alone or in combination with BAF were also double stained with Annexin V-FITC and PI. The percentage of positive cells determined over 10000 events was analysed on a FACScan cytofluorimeter using the CellQuest software.

### AVO detection

5637 and T24 BC cells (3×10^4^/ml) were seeded into 24-well plates and treated or not with CPS (300 μM) for 48 h. Cells were then washed with medium, stained with AO (1μg/ml) for 15 min and analyzed on a FACScan cytofluorimeter using the FL1 and FL3 fluorescence. The percentage of positive cells determined over 10000 events was evaluated using the CellQuest software.

### Western blot analysis

5637 and T24 cells, untreated or treated CPS (300 μM) for different times, were lysed in a lysis-buffer containing protease inhibitor cocktail (Sigma Aldrich). Lysates were separated on sodium dodecyl sulphate polyacrylamide gel (9 and 14%) and transferred. After blocking with 5% low-fat dry milk in phosphate-buffered saline (PBS) 0.1% Tween 20 for l h, blots were incubated with the primary Abs: anti-LC3, anti-p62, anti-AMPK, anti-pAMPK, anti-Atg4C, or anti-GAPDH, followed by the appropriate HRP-conjugated antibodies. To assess the autophagic flux, cells were treated with CPS alone or in combination with BAF (25 nM). The LC3 conversion was also assessed in BC cells treated for 72 h with CPS (300 μM) alone or in combination with NAC (10 mM). In addition, western blot was performed in lysates from siGLO or siBeclin 1 BC cells, treated or not with CPS (300 μM) for 72 h, using anti-Beclin 1 or anti-LC3 Abs, to evaluate silencing efficacy and autophagy respectively. Moreover LC3 levels were investigated in lysates from siGLO or siPTCH2 BC cells treated, as above described, with CPS. The detection was performed using LiteAblot Turbo (EuroClone, Milano, Italy) and densitometric analysis was carried out evaluating three independent experiments by Chemi-Doc using Quantity One software (Bio-Rad, CA, USA). GAPDH levels were used as loading control.

### ROS production

5637 and T24 BC cells (3×10^4^/ml) were seeded into 24-well plates and cultured for 4, 8, 12, 20 and 24 h with CPS (300 μM) or vehicle. Cells were washed with PBS, pulsed with DCFDA for 10 min at 37°C, 5% CO_2_, and analyzed by FACScan cytofluorimeter using the CellQuest software. In some experiments, ROS production was assessed in BC cells treated for 24 h with CPS (300 μM) alone or in combination with NAC (10 mM), ROT (1μM) and DPI (0.1 μM).

### Mitochondrial transmembrane potential (ΔΨm)

ΔΨm was evaluated by JC-1 staining. 5637 and T24 BC cells (3×10^4^/ml), seeded into 24-well plates, were treated with CPS (300 μM) or vehicle for different times (0, 6, 8, 12 and 24 h) and then incubated for 10 min at room temperature with JC-1. JC-1 was excited by an argon laser (488 nm); green (530 nrn)/red (>570 nrn) emission fluorescence was collected simultaneously. Carbonyl cyanide chlorophenylhydrazone protonophore, a mitochondrial uncoupler was used as positive control (data not shown). Samples were analyzed by a FACScan cytofluorimeter using the CellQuest software; fluorescence intensity was expressed in arbitrary units on logarithmic scale.

### Measurement of ADP/ATP ratio

5637 and T24 BC cells (3×10^4^/ml) were seeded into 96-well plates and cultured with CPS (300 μM) for 12 h at 37°C, 5% CO_2_. At the end of treatment, ADP/ATP ratio was measured by the EnzyLight ADP/ATP Ratio Assay Kit (BioAssay Systems, CA, USA) following the instructions. Bioluminescence was acquired by FluoStar OMEGA luminometer (BMG LABTECH GmbH, Ortenberg, Germany).

### Gene expression analysis

Total RNA was extracted from 5637 and T24 BC cells, treated for 12 h with CPS (300 μM) or vehicle, with the RNeasy Mini Kit (Qiagen) and reverse transcribed using the Reaction Ready first strand cDNA kit (Superarray Bioscience Corporation, MD, USA). Quantitative Real Time PCR (qRT–PCR) was performed using the IQ5 Multicolor Real-time PCR detection system (Bio-Rad), the RT2 real-time SYBR green PCR Mix and the Human Autophagy plates according to the protocol (Qiagen).

In addition, total RNA from BC cells, untreated or treated with CPS (300 μM) for 120 h, was extracted with the RNeasy Mini Kit (Qiagen), and cDNA was synthesized using the High-Capacity cDNA Archive Kit (Applied Biosystems, PA, USA) according to the manufacturer's instructions. The EMT pathway was analysed by qRT–PCR using a Custom Taqman assay (Thermofisher Scientific, MA, USA). Moreover, to evaluate the efficacy of PTCH2 silencing, qRT–PCR was performed using ddPCR GEX Assay for PTCH2 and GAPDH (Bio-Rad) and the QuantiFast Multiplex PCR Master Mix (Qiagen).

### Fluorescence-activated cell sorting analysis

5637 and T24 BC cells, plated in 24 well plate at the concentration of 3×10^4^/ml, were treated with vehicle or CPS (300 μM) for 120 h. Then cells were detached, counted and fixed with 4% paraformaldehyde for 10 min at room temperature. After washing in staining buffer (0.1% sodium azide, 1% FBS in PBS) cells were stained with anti-β_1_ and anti-α_5_ integrin subunits followed by FITC-conjugated secondary Ab. In addition BC cells were also permeabilized using Perm solution (1% saponin, 0.1% sodium azide, 1% FBS in PBS) and labelled with anti-E cadherin, anti-vimentin, anti-ILK and anti-Bcl2 followed by respective FITC-conjugated secondary Abs. In some experiments, the expression of vimentin and α_5_ integrin subunit was valuated in siGLO and siPTCH2 cells after treatment for 120 h with CPS. Samples were analysed by a FACScan cytofluorimeter using the CellQuest software (Becton Dickinson).

### Morphological analysis

5637 and T24 BC cells, plated in 24 well plate at the concentration of 3×10^4^/ml, were treated with vehicle or CPS (300 μM) for 120 h. Then morphological analysis was assessed by citofluorimetric analysis using forward scatter parameter and by light microscope at X40 magnification using a BX51 microscope (Olympus, Milan, Italy).

### BrdU incorporation

5637 and T24 BC cells, plated in 24 well plate at the concentration of 3×10^4^/ml, were treated with CPS (300 μM) for 120 h. Then cells were detached, counted and plated at the concentration of 3×10^4^/ml in a 6 well plate. The day after, BrdU (20 μM) was added for additional 24 h. At the end of the BrdU incorporation BC cells were fixed using 70% ice-cold ethanol and stained with anti BrdU Ab (1:20) followed by FITC-conjugated secondary Ab (1:40). Samples were analysed by a FACScan cytofluorimeter using the CellQuest software. Fluorescence intensity was expressed in arbitrary units on logarithmic scale.

### Statistical analysis

The statistical significance was determined by Student's t-test and Anova. No statistical significant difference was found between untreated and vehicle (DMSO)-treated cells or comparing different times of vehicle-treatment each other.

## SUPPLEMENTARY MATERIALS FIGURE


