# Supplementary material for: Capsaicin triggers autophagic cell survival which drives epithelial mesenchymal transition and chemoresistance in bladder cancer cells in an Hedgehog-dependent manner
Source: Oncotarget. 2016 Jun 29;7(31):50180–94. doi: 10.18632/oncotarget.10326 (PMC5226576; doi:10.18632/oncotarget.10326)
Supplement: Supplementary file 1 [file oncotarget-07-50180-s001.pdf]

Capsaicin triggers autophagic cell survival which drives epithelial mesenchymal transition and chemoresistance in bladder cancer cells in an Hedgehog-dependent manner

SUPPLEMENTARY FIGURE

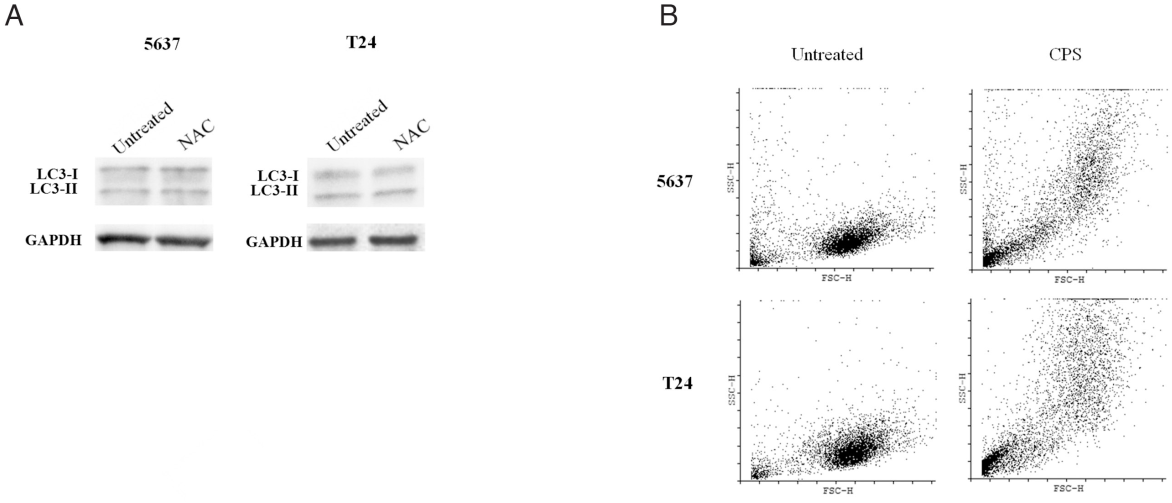

Supplementary Figure S1: A. Lysates from BC cells untreated or treated for 72h with NAC (10 mM) were separated on 14% SDS-PAGE and probed with anti-LC3 Ab. Cropped blots are representative of one of three separate experiments. GAPDH protein levels were used as loading control. **B.** Cell size was evaluated in BC cells, untreated or treated with CPS (300 µM) for 120 h by FACS analysis using forward scatter parameter. One representative out of three independent experiments is shown.
